# Chitosan-Sulfated Titania Composite Membranes with Potential Applications in Fuel Cell: Influence of Cross-Linker Nature

**DOI:** 10.3390/polym12051125

**Published:** 2020-05-14

**Authors:** Andra-Cristina Humelnicu, Petrisor Samoila, Mihai Asandulesa, Corneliu Cojocaru, Adrian Bele, Adriana T. Marinoiu, Ada Sacca, Valeria Harabagiu

**Affiliations:** 1 “Petru Poni” Institute of Macromolecular Chemistry, Aleea Grigore Ghica Voda 41A, 700487 Iasi, Romania; humelnicu.andra@icmpp.ro (A.-C.H.); asandulesa.mihai@icmpp.ro (M.A.); cojocaru.corneliu@icmpp.ro (C.C.); bele.adrian@icmpp.ro (A.B.); 2 National Research and Development Institute for Cryogenics and Isotopic Technologies – ICSI Rm. Valcea, 240050 Ramnicu Valcea, Romania; adriana.marinoiu@icsi.ro; 3 National Research Council of Italy, Institute for Advanced Energy Technologies “Nicola Giordano” (CNR-ITAE), via S. Lucia sopra Contesse 5, 98126 Messina, Italy; ada.sacca@itae.cnr.it

**Keywords:** chitosan, sulfated titania, cross-linking, polyelectrolyte composite membranes

## Abstract

Chitosan-sulfated titania composite membranes were prepared, characterized, and evaluated for potential application as polymer electrolyte membranes. To improve the chemical stability, the membranes were cross-linked using sulfuric acid, pentasodium triphosphate, and epoxy-terminated polydimethylsiloxane. Differences in membranes’ structure, thickness, morphology, mechanical, and thermal properties prior and after cross-linking reactions were evaluated. Membranes’ water uptake capacities and their chemical stability in Fenton reagent were also studied. As proved by dielectric spectroscopy, the conductivity strongly depends on cross-linker nature and on hydration state of membranes. The most encouraging results were obtained for the chitosan-sulfated titania membrane cross-linked with sulfuric acid. This hydrated membrane attained values of proton conductivity of 1.1 × 10^−3^ S/cm and 6.2 × 10^−3^ S/cm, as determined at 60 °C by dielectric spectroscopy and the four-probes method, respectively.

## 1. Introduction

Polymer electrolyte membranes (PEMs) play a key role in fuel cells as they transport charges between electrodes and prevent fuel leaks [1,2]. Nafion^TM^ PEMs are recognized for their high proton conductivity, good mechanical properties, and chemical and electrochemical stability [3]. However, Nafion membranes are extremely expensive, their preparation and use are hazardous for the environment, and they are also characterized by fuel crossover and low durability [4,5,6]. Other shortcomings of these membranes consist in water management problems, need for a full humidification since their high proton conductivity is strictly linked to the H_3_O^+^ hydration, and, as a consequence, the optimal operative temperature is limited below 100 °C [7].

In this respect, biopolymer-based membranes are more and more considered as strong candidates to develop cheaper and more environmentally friendly PEMs. Recently, chitosan membranes were proposed as substitutes Nafion PEMs [8,9,10]. In spite of promising results, there is still research effort required to improve the mechanical strength and the conductivity of chitosan membranes [5]. The solutions often proposed were related to their doping with inorganic fillers (e.g., solid superacids known for their high proton conductivities and hygroscopic properties) [11] and/or chemical modification of the polysaccharide by chemical cross-linking [12]. The cross-linking is the one of the most efficient ways to improve the characteristics of pristine chitosan membranes, by ameliorating their thermal and mechanical properties, as well as their water uptake capacities, with direct impact on the conductivity increasing [12]. However, to the best of our knowledge, little attention was paid on the influence of the cross-linker nature on the properties of chitosan-solid superacid hybrid PEMs.

The objective of this study was to develop novel chitosan-based composite membranes and to follow their structure, morphology, mechanical, and thermal properties, as a function of the cross-linker nature. To this end, we selected sulfated titanium dioxide as inorganic filler and sulfuric acid, pentasodium triphosphate and epoxy-terminated polydimethylsiloxane as cross-linkers, the last one not reported for the preparation of PEMs considered for fuel cell application. Typical tests used to describe PEM performances—such as water uptake, chemical stability and conductivity behavior—were also carried out.

## 2. Materials and Methods 

### 2.1. Materials 

Chitosan (CS) with molecular weight of 290 kDa (determined by a viscometric study and according to Equation (S1) on Supplementary Materials) and 82% degree of deacetylation (determined by ^1^H NMR (Figure S1 on Supplementary Materials), TiO_2_ (anatase, particle size < 25 nm, 99.7% purity), bis(glycidyloxypropyl)-terminated polydimethylsiloxane of *M*_n_ = 980 Da (PDMS), acetic acid, sulfuric acid 98%, pentasodium triphosphate (TPP) and sodium hydroxide were purchased from Sigma Aldrich (Taufkirchen, Germany). Methanol (purris p.a.) was supplied by Chemical Company (Iasi, Romania). All reagents were of analytical grade and used without further purification.

Sulfated TiO_2_ (TS) was obtained by adapting the methods described by Li et al. [13] and Ayyaru et al. [14]. For this purpose, 22.5 mg of TiO_2_ nanoparticles were dispersed in a solution containing 10 mL of methanol and 5 mL of 1 M H_2_SO_4_ through sonication for 30 min in a Emmi 12HC bath. The suspension was then centrifuged, washed with water, and the solid was dried in oven at 105 °C for 6 h to obtain the sulfated TiO_2_ sample (TS) with a content of 0.354 mmol sulfate groups/g (evaluated according to Equation (S2) on Supplementary Materials).

### 2.2. Preparation of Membranes

#### 2.2.1. Preparation of Chitosan-Sulfated Titania Composite Membranes (CS-TS)

CS-TS composite membranes were prepared using 5 wt % of sulfated TiO_2_ relative to the amount of chitosan. In the first step, a stock solution of chitosan (3% w/v) was prepared by dissolving the polysaccharide in 2% acetic acid solution. Subsequently, precisely determined amount of TS was dispersed in 5 mL water, sonicated for 15 min and added over 15 mL of chitosan solution, under continuous stirring at 600 rpm for 30 min. The final mixture was poured into 9.6 cm diameter Petri dish and dried at 30 °C for 48 h (up to constant weight) to obtain CS-TS membrane.

#### 2.2.2. Membrane Cross-Linking by Sulfuric Acid (HS) and by Pentasodium Tripolyphosphate (TPP)

The cross-linking was carried out by adapting already published procedures. Thus, dried CS-TS membranes were immersed into 1 M H_2_SO_4_ solution (pH = 0.38) for 15 min [15] or in 2% TPP for 2 h solution (pH = 8.64) [16]. Subsequently, the membranes were washed with distilled water and dried at room temperature for 48 h until constant weight was achieved. The resulted composite membranes, with average thicknesses of about 70 μm (from SEM images of the cross-sections), were labeled as CS-TS-HS and CS-TS-TPP, respectively.

#### 2.2.3. Cross-Linking by Bis(glycidyloxypropyl)-Terminated Polydimethylsiloxane (PDMS) 

The PDMS cross-linked composite membrane (CS-TS-PDMS) was obtained adapting a procedure previously described for the preparation of PDMS modified chitosan [17]. Shortly, PDMS (NH_2_/epoxy = 1/1 molar ratio) was added in situ to the CS/TS dispersion prepared as described in the previous paragraph and the mixture was stirred at 40 °C until complete homogenization. After casting into Petri dishes and oven drying at 30 °C, the obtained membrane was washed with distilled water and dried at room temperature for 48 h until constant weight was attained. The average thickness of the dried membrane was of around 130 μm, as determined from SEM image of the membrane cross-section. 

### 2.3. Materials Characterization

Structural characterization of TS and pristine chitosan (CS) intermediates as well as of the composite membranes was performed by FTIR spectroscopy using a Bruker Vertex 70 spectrometer (Bruker Optics, Ettlingen, Germany) and KBr pellet method. The surface and cross-section morphology and elemental composition of the composite membranes were investigated by scanning electron microscopy using an (ESCM) Quanta 200 device (SEM, FEI Company, Brno, Czech Republic)) coupled with energy dispersive X-ray (EDX) system (EDAX, Mahwah, NJ, USA). The cross-sections were obtained by tearing the liquid nitrogen frozen membranes.

The thermal properties of the samples were studied under nitrogen atmosphere in the temperature range of 20–700 °C, with a heating rate of 10 °C/min on a Jupiter thermal analysis system TG-DSC Model STA449F1 (NETZSCH, Selb, Germany). The strain-stress curves were obtained by using a two-column Instron Model 3365 device equipped with a 500 N cell force (Instron, Norwood, MA, USA). In this respect, dumb-bell shaped samples (*L* = 5 cm; *l* = 4 mm; active length = 3.5 cm) were cut using a press and were tested for uniaxial stress–strain curves with a 50 mm/min elongation speed.

The kinetics of water uptake was evaluated at 25, 60 and 80 °C. In this respect, 1 cm^2^ of membrane samples were dried until constant weight (*W*_dry_). Subsequently, the samples were immersed in 30 mL distilled water at different temperatures. At regular time intervals, the membranes were extracted, the water excess was removed by buffering the samples on filter paper and weighting (*W*_wet_). The water uptake (*WU*%) was calculated for each sample using the formula

(1) $$WU\left(\%\right)=\left(\left(W_{\mathrm{wet}}-W_{\mathrm{dry}}\right)/W_{\mathrm{dry}}\right)\times 100$$

The oxidative stability of the membranes was studied by immersing 1 cm^2^ dry samples in 10 mL of freshly prepared Fenton reagent (3 vol % H_2_O_2_ solution containing 4 ppm Fe(SO_4_)_2_·7H_2_O) at room temperature. The membranes were extracted from the solution after 1 h or after 24 h, dried and weighed in order to determine the weight loss of the sample as a function of time. 

Dielectric spectroscopy measurements were performed with a broadband dielectric spectrometer (Novocontrol, Montabaur, Germany) equipped with a high-resolution Alpha-A analyzer and a Quatro Cryosystem temperature controller. Complex dielectric permittivity spectra were recorded under isothermal conditions by applying an alternating electrical field of 1 V in a broad range of frequency (0.1–10^7^ Hz). The composite membranes were sandwiched between two gold-plated flat electrodes and measurements were carried out under pure nitrogen, preventing the moisture from environment. The measurements were performed on dry (samples kept at 80 °C into a vacuum oven for 12 h) and hydrated membranes (obtained by immersing the samples in distilled water for 1 h at room temperature prior to dielectric spectroscopy measurements). The dielectric spectra were collected in steps of 5 °C with 0.1 °C stability and high reproducibility, at temperatures from 0 to 160 °C and from 0 to 80 °C for dry and hydrated sample, respectively.

The proton conductivity (PC) measurement on CS-TS-HS membrane was carried out in the longitudinal direction (in-plane) by the four-probes method at two different temperatures (30 and 60 °C), at fully humidification level (100% RH), P = 1 atm, using a hydrogen flux of 1000 sccm, as suggested by the supplier, and DC current by using a PTFE commercial BT-112 Bekktech conductivity cell (Bekktech, LLC acquired by Scribner Associates Inc. in 2011, Southern Pines, NC, USA) with a 5 cm^2^ fixture hardware by Fuel Cell Technologies, Inc. (BekkTech product no. ACC-920). The cell was connected to a test station and a potentiostat-galvanostat (AMEL mod.551) [18,19]. A membrane sample of about 2.5 × 0.52 cm^2^ was cut by a sample punch (BekkTech product no. ACC-960) and its size was measured through a width measurement tool (BekkTech product no. ACC-940) with a magnification of 11× and a reticule with 0.1 mm gradients. The thickness was measured by a Mitutoyo electronic gauge. The membrane was assembled in the cell and placed in contact with the two fixed platinum electrodes. By an indirect imposition of the current, a voltage drop between the two fixed electrodes was measured. The electrical resistance values were obtained by extrapolating the data from the plot of current as a potential function. At the end, the PC (σ, S·cm^−1^) was calculated using the formula

(2) σ=L/R·W·T

where *L* = 0.425 cm, fixed distance between the two Pt electrodes; *R* = resistance in Ω; *W* = sample width in cm; *T* = sample thickness in cm. The measurement and cell set-up are detailed in Figure S2.

## 3. Results and Discussions

### 3.1. Membrane Preparation

**Scheme 1:**
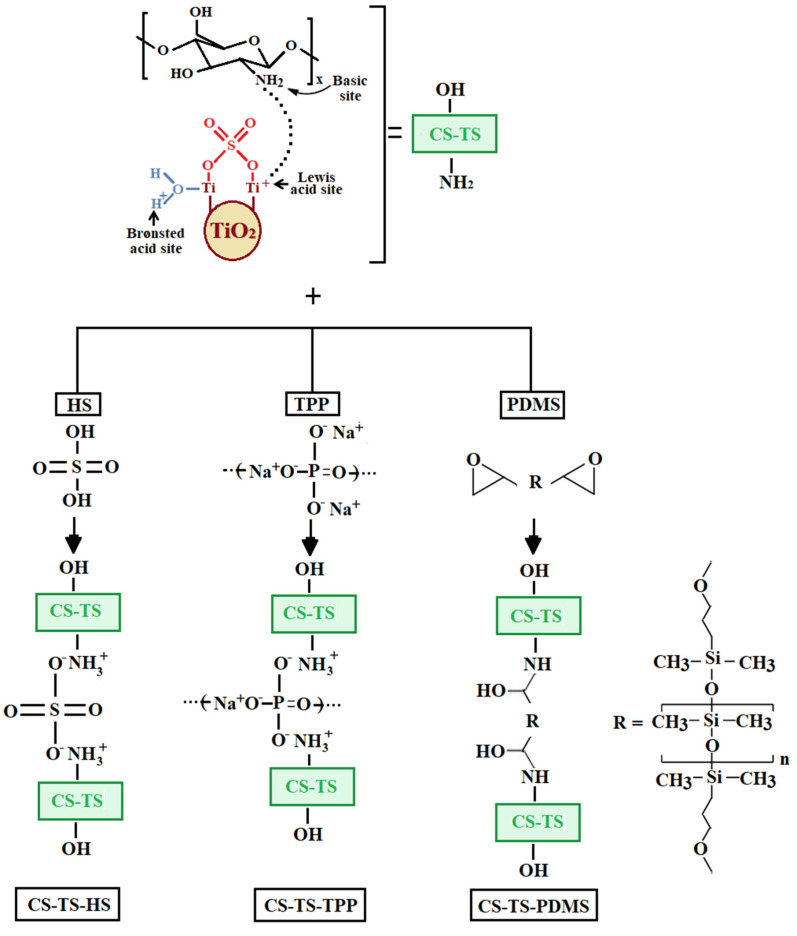
Preparation of CS-based composite membranes.

CS-TS membrane of a content of 5 wt % TS filler was prepared by simple mixing the components and drying. Ionic interactions between Lewis acid sites on filler surface and amino basic groups of CS were proposed to stabilize the inorganic particles into CS matrix [13] (Scheme 1). 

Three different agents, such as sulfuric acid (HS), pentasodium tripolyphosphate (TPP), and bis(glycidoxypropyl)-terminated polydimethylsiloxane (PDMS) were further used for cross-linking and to study their influence on the structure and the properties of the composite membranes. The first two cross-linkers provide a well-known electrostatic interactions between protonated amino groups of CS and cross-linker anions [15], while PDMS undergoes covalent cross-linking through the well-known reaction between CS amino groups and epoxy units attached to the siloxane chains [17] (Scheme 1). Based on its intrinsic properties (high hydrophobicity, very low *T*_g_, thermal stability up to 300 °C, relatively low variation of its properties with temperature [20], PDMS cross-linker is expected to provide to the membrane a higher flexibility and lower shrinkage during drying, as well as a controllable hydrophilic–hydrophobic balance.

### 3.2. FTIR Characterization

**Figure 1:**
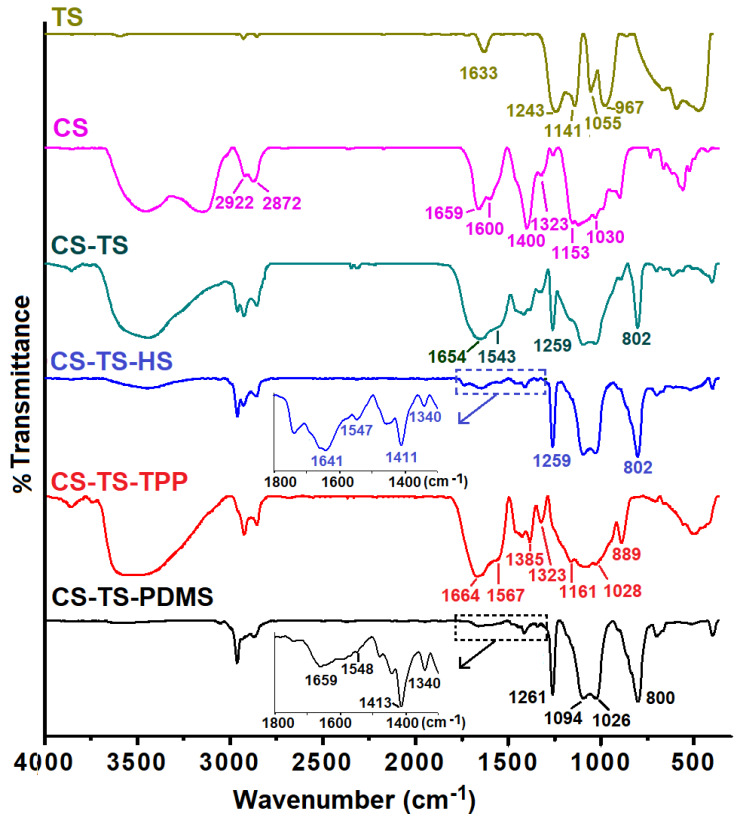
FTIR spectra of sulfated TiO_2_ (TS), pristine chitosan (CS) and CS-TS, CS-TS-HS, CS-TS-TPP, and CS-TS-PDMS composite membranes.

The structure of the prepared materials was first assessed through FTIR spectroscopy. Figure 1 compares the normalized FTIR spectra of TS and CS intermediates with those of the composite membranes. Apart from the absorption band at 1633 cm^−1^ (deformation vibration, adsorbed water) and of a broad band in the range of 879–409 cm^−1^ (Ti–O), in the spectrum of TS, the presence of four other absorption bands between 1243 and 967 cm^−1^ (1243 and 1141 cm^−1^, asymmetric and symmetric stretching vibrations of S=O groups; 1055 cm^−1^ and 967 bands, asymmetric and symmetric stretching vibrations of S–O units) confirms the sulfating process and indicates a bidentate coordination of sulfate groups to Ti atoms, as previously stated [13,21].

For CS sample the characteristic absorption bands appear in the range 4000–800 cm^−1^ [22]. Thus, broad bands between 3500–3100 cm^−1^ (O–H and N–H stretching vibration) are visible in the CS spectrum, while asymmetric and symmetric stretching vibrations of C–H groups are located at 2922 and 2872 cm^−1^, respectively. Chitosan shows also characteristic bands at 1659 cm^−1^ (C=O, amide I), 1600 cm^−1^ (N–H in plane deformation, primary amine), 1400 cm^−1^ (N–H deformation, amide II) and 1323 cm^−1^ (C–N stretching vibrations, amide III). In the range 1153–1030 cm^−1^ the bands were attributed to C–O–C groups [23]. 

Modified FTIR spectra as compared to those of the pristine TS and CS components are observed for the composite membranes confirming the presence of these components in the membrane structure and the interaction of the cross-linker with chitosan matrix.

Thus, the presence of acidic groups determines the protonation of amino groups of chitosan and the shifting of the primary amine absorbance from 1600 cm^−1^ in CS spectrum to 1543 and 1547 cm^−1^, respectively in CS-TS and CS-TS-HS spectra (see insert) [15]. Moreover, the interaction of sulfate groups with chitosan determines shifting of amide I and amide III bands to lower (decreased bond strength), respectively higher wavenumbers (increased bond strength). Both composite membranes also show strong bands attributed to the sulfate (1259 cm^−1^) and to Ti–O–Ti vibrations (superposed on the NH_3_^+^ rocking vibrations [24,25] and located at 802 cm^−1^).

The successful cross-linking of CS-TS membrane with TPP is confirmed by the shifting of the primary amine absorbance to 1567 cm^−1^ due to its protonation and interaction with the phosphate groups as well as the presence of the P–O–P bridge asymmetric stretching vibration at 889 cm^−1^ [26]. The other phosphate characteristic bands are overlapped on C–O–C vibrations of CS in the range of 1161–1028 cm^−1^ and different effect of phosphate groups on amide bands as compared to that of sulfate groups is evidenced, i.e., the increase and decrease of amide I, amide II bonds strength respectively, while amide III absorption remain unchanged. 

In the FTIR spectrum of the CS-TS-PDMS membrane, the covalent linking through the reaction of amino groups of CS and epoxy units attached to the siloxane chains, as well as the increasing of membrane hydrophobic character induced by siloxane component determines a notable diminishing of the absorbance bands between 3500–3100 cm^−1^. Moreover, strong bands characteristic to siloxane moiety (1261 and 800 cm^−1^ Si–CH_3_; 1094–1026 cm^−1^, Si–O–Si) [17], partially covering the bands of CS are also visible in the spectrum. 

One should also mention the diminishing of the characteristic bands of CS in the region 1670–1300 cm^−1^ for both CS-TS-HS and CS-TS-PDMS samples due to the effect of the strong Si–O–Si (1100–1000 cm^−1^) and sulfate (1259 cm^−1^) bands on shorter bands in normalized spectra. (see inserts).

### 3.3. SEM Characterization 

**Figure 2:**
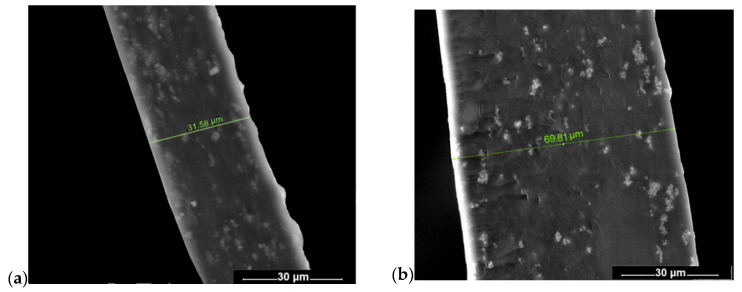

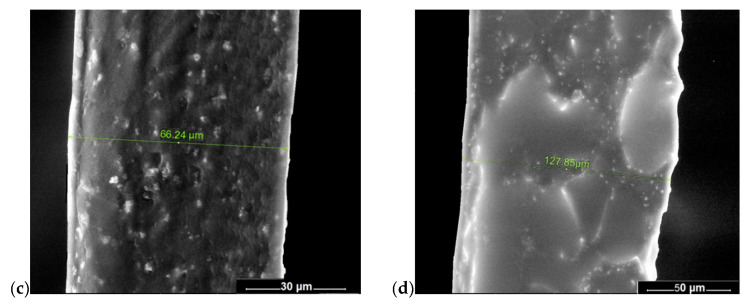
Representative cross-section SEM images of (**a**) CS-TS, (**b**) CS-TS-HS, (**c**) CS-TS-TPP, and (**d**) CS-TS-PDMS composite membranes.

**Figure 3:**
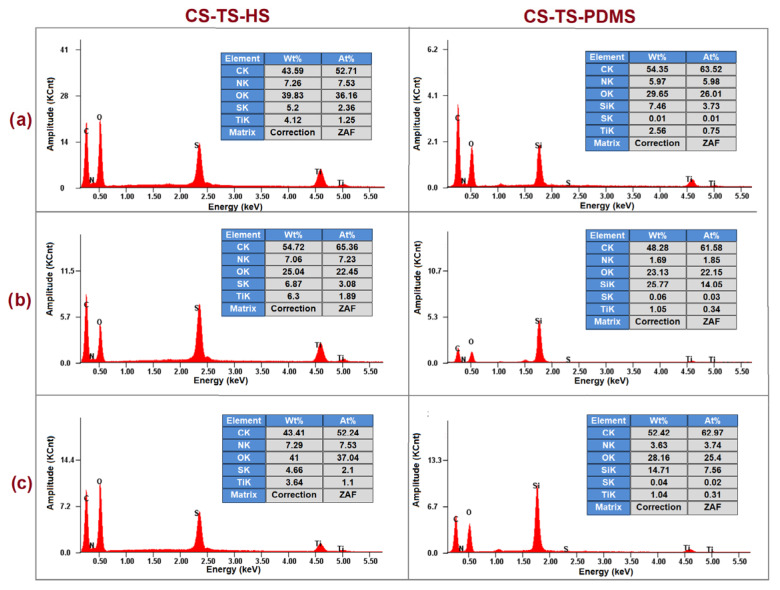
EDX spectra and elemental compositions of CS-TS-HS (**left**) and CS-TS-PDMS (**right**) composite membranes (**a**) polymer/glass interface, (**b**) cross-section, and (**c**) polymer/air interface.

Representative SEM images of membranes recorded for membrane cross-sections are shown in Figure 2. From the analysis of SEM micrographs, one may observe that all prepared membranes are dense, with no detectable interconnected pores. TiO_2_ nanoparticles are quite well dispersed throughout the cross-section of membranes for all studied materials. In addition, CS-TS-PDMS membrane shows a phase separated morphology, with distinct domains at micro scale level, as a consequence of strong incompatibility between hydrophilic chitosan and hydrophobic polysiloxanes sequences [20]. Otherwise, according to the literature, siloxanes polymers tend to migrate at the surface exposed to air [27]. In this respect, elemental analysis was performed on CS-TS-PDMS membrane surfaces and in the cross-section (Figure 3). The EDX data confirm the presence of Si in higher concentrations at air exposed interface compared to polymer/glass interface. Nevertheless, one may notice that most of Si atoms are mainly concentrated in cross-section.

As one may also see from Figure 2, the thicknesses of the cross-linked membranes (about 70 µm for CS-TS-HS and CS-TS-TPP samples and about 130 µm for CS-TS-PDMS membrane) are larger as compared to CS-TS pristine membrane (about 30 µm), due to the incorporation of the cross-linking agents. Similar results were obtained on poly(styrene–2-vinylpyrridine) films crosslinked by quaternization of pyridine units with diiodobutane [28]. The SEM micrographs recorded for the membrane surfaces (Figure S3), confirm that all the obtained membranes are dense, with uniform dispersion of TiO_2_.

EDX analysis for CS-TS-HS and CS-TS-PDMS Figure 3) also showed an average content of nitrogen of 7.2%, respectively 3.7%, confirming the presence of CS in these samples that showed small amide absorptions in their FTIR spectra.

### 3.4. Thermogravimetric Analysis

**Figure 4:**
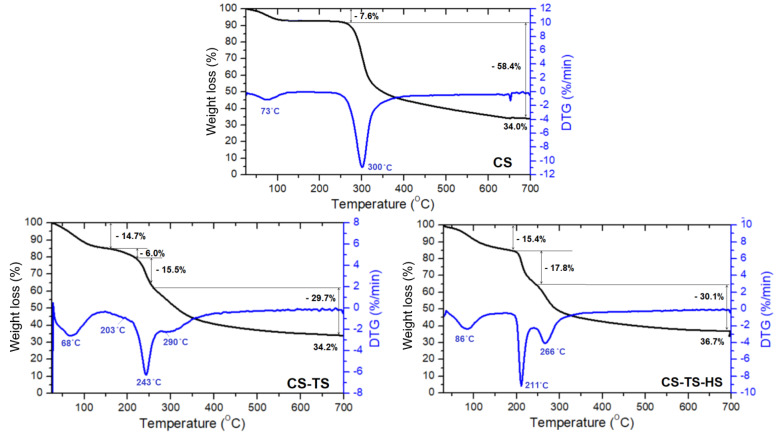

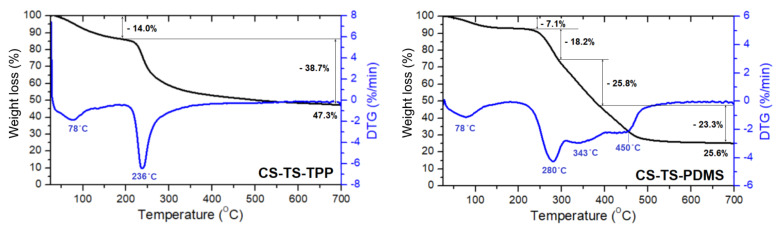
TG/DTG curves of CS-TS, CS-TS-HS, CS-TS-TPP, and CS-TS-PDMS composite membranes compared with pristine chitosan (CS).

Figure 4 presents the thermogravimetric (TG) and thermogravimetric derivative (DTG) curves for pristine CS and composite membranes registered in nitrogen atmosphere, between 20 and 700 °C. CS degrades through a complex mechanism involving deacetylation followed by dehydration, deamination, and depolymerization processes. At temperatures lower than 300 °C, two main weight losses are noticed, centered at *T*_max_ = 73 °C and 300 °C, respectively, as also reported previously by others [29]. The first stage (*T*_max_ = 73 °C) is attributed to the evaporation of water and residual acetic acid solvent and the second stage (*T*_max_ = 300 °C) is concerned with degradation of chitosan chains. At temperatures higher than 300 °C, the sample continuously degrades up to a residual percentage weight of 34% at 700 °C.

The thermal decomposition of composite membranes strongly depends on the nature of cross-linking agent. Similarly to CS, all of them showed water solvent and acetic acid traces evaporation phenomena with *T*_max_ between 56 and 86 °C. The presence of acidic groups either linked to TS or coming from HS and TPP cross-linkers reduces the stability of all composite membranes, the second decomposition *T*_max_ being about 20–90 °C lower than the value corresponding to pristine CS. However, comparable weight losses at 700 °C are observed for CS, CS-TS and CS-TS-HS samples, while lower and higher weight losses are registered for CS-TS-TPP and CS-TS-PDMS membranes, respectively. Moreover, multi-stage decomposition behavior is observed for composite membranes.

CS-TS membrane shows the second major weight loss with two maxima at 203 °C, attributed to the loss of sulfate groups, and 243 °C, attributed to the decomposition of amorphous part of CS matrix. The CS more crystalline domains are decomposing at slightly lower *T*_max_ (290 °C) as compared to pristine CS. CS-TS-HS membrane presents a second stage of decomposition at *T*_max_ = 211 °C (sulfate groups) and a third one (CS chains) at *T*_max_ = 266 °C due to the action of higher amounts of sulfate groups on the polysaccharide structure. CS-TS-TPP membrane showed only a second stage degradation with *T*_max_ of 237 °C. One may notice that this step occurs at a temperature close to the loss of the sulfate groups. On the other hand, literature data indicate that TPP cross-linked chitosan degrades at lower temperatures than pure chitosan (around 230 °C) and this behavior is explained by a decrease in crystallinity of the polysaccharide following crosslinking with TPP [30]. Thus, at this stage, the degradation of both sulfate groups and polymer occurs. Apart the first step of water evaporation, the thermal degradation of the CS-TS-PDMS sample is more complex and involves simultaneous decomposition of the functional sulfate, hydroxyl and alkyl amines units (at 280 °C) and of CS and PDMS chains at 342 and 450 °C, respectively Note that all prepared membranes are thermally stable to relatively high temperatures (above 200 °C).

### 3.5. Mechanical Properties

**Figure 5:**
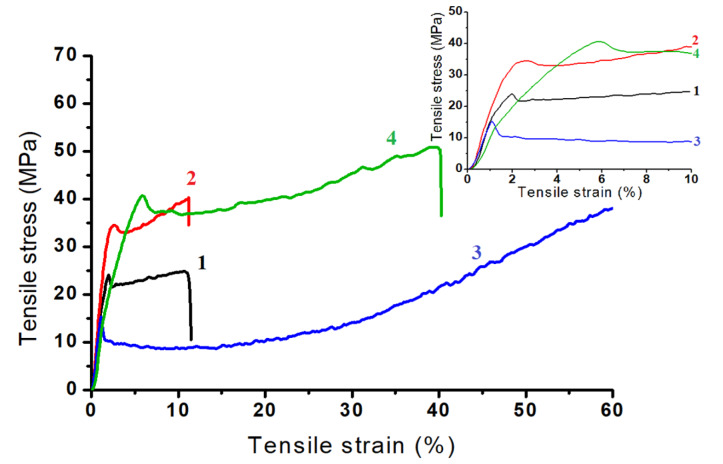
Mechanical stress–strain profiles at high and low (insert) tensile strain values of CS-TS (**1**), CS-TS-HS (**2**), CS-TS-TPP (**3**), and CS-TS-PDMS (**4**) composite membranes.

To study the mechanical properties of the produced membranes, typical mechanical tests were performed by recording the stress–strain profiles (Figure 5). The mechanical properties were ascertained in terms of fracture strain, tensile stress, and Young’s modulus (Table 1). 

As expected, membrane mechanical properties were influenced by the cross-linker nature. All samples present stress–strain curves specific to plastic materials with a clear elastic domain at low strains, followed by a yield strength and plastic deformation. Due to the nature of the siloxane bond (highly flexible), the yield strength of CS-TS-PDMS shifted to larger strains. Moreover, the cross-linking of CS-TS membrane with TPP resulted in a tougher material, since no strain break occurred. These observations were corroborated by the data presented in Table 1.

The cross-linking process of composite membranes affects the calculated values for fracture strain, tensile stress, and Young’s modulus. Thus, compared to CS-TS sample, one may observe that the elongation at break is higher for the cross-linked membranes. It should be noted that the same trends were found for the tensile stress values, except for CS-TS-TPP sample. One may also notice that the cross-linking processes decrease Young’s modulus values whatever the cross-linking agent, except the membrane cross-linked with sulfuric acid, but its values are higher than 1 GPa for all samples.

### 3.6. Water Uptake Capacity of Composite Membranes

Sufficient water content is essential in the operation of fuel cells determining the membrane performance, stability, and durability [31]. As expected, CS-TS sample was completely dissolved in water after less than 5 min. Water uptake kinetics curves of cross-linked membranes are given in Supplementary Materials (Figure S4) and the values observed after 24 h are listed in Table 1. All cross-linked membranes revealed very high water uptake capacities for all temperatures considered, with values ranging from 88% (for amphiphilic CS-TS-PDMS sample at 60 °C) to 184% (for CS-TS-HS sample at 80 °C). Moreover, water uptake mainly occurred in the first minutes of experiment. Generally, the cross-linked membranes exhibit superior capabilities at 25 °C compared to 60 and 80 °C. These findings are in good agreement with the literature and confirm that the moisture adsorbed by chitosan films decrease with the increase of temperature [32].

### 3.7. Chemical Stability of Composite Membranes

The oxidative stability of membranes are often used in the evaluation of PEMs [33]. In this respect, the membranes were challenged with freshly prepared Fenton’s reagent (Table 1). The unmodified membrane (CS-TS) was completely dissolved in Fenton’s reagent, while the cross-linked membranes were relatively stable. The resistance to oxidation of the membranes decreased in 24 h from weight loss values of up to 10 wt % in 1 h, to 15, 22, and 30 wt % for the samples cross-linked with HS, TPP, and PDMS, respectively, the CS-TS-HS membrane showing the best oxidation resistance. 

### 3.8. Broadband Dielectric Spectroscopy

#### 3.8.1. Overall Dielectric Behavior of Dry Membranes

**Figure 6:**
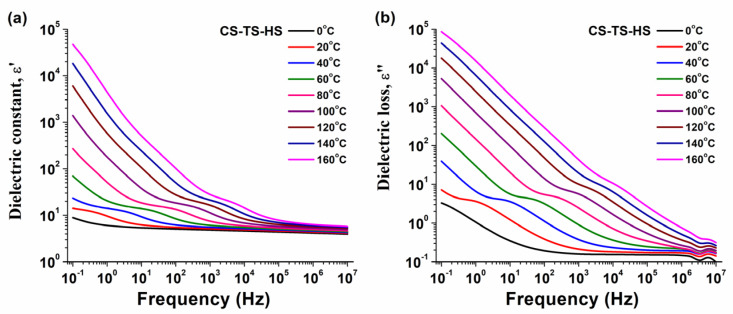
Evolution of the dielectric constant (**a**) and of the dielectric loss with frequency (**b**) for dry CS-TS-HS composite membrane.

Figure 6 displays the evolution of dielectric constant (ε’) and dielectric loss (ε”) with frequency for dry CS-TS-HS membrane, as a representative example. The spectra corresponding to dry CS-TS, CS-TS-TPP, and CS-TS-PDMS samples are found on Supplementary Materials (Figure S5). 

As is generally known, the dielectric constant is related to the orientation of chemical dipoles in the direction of an alternating electrical field. ε’ diminishes gradually with increasing frequency, since the dipoles can no longer follow the oscillations of alternative field [34]. According to Figure 6a, the CS-TS-HS membrane provided a high dielectric constant in the considered integral frequency range, revealing an intense dipolar activity. The strong decrease of the dielectric constant, especially at low frequencies is an effect of ionic polarization induced by the sulfate groups [34]. Moreover, the magnitude of ε’ increased with temperature due to increased mobility of polymer segments having a dipole moment.

The dielectric loss parameter comprises the dissipated energy for the dipole alignment motions and the energy required to move ions in response to the alternating electrical field. As a consequence, both the polarization and the electrical conductivity signals are observed. In Figure 6b, the dielectric loss curves revealed a linear evolution with frequency, especially at low frequencies and high temperatures, with a slope close to −1, which is characteristic for the ionic conductivity-type signal. In fact, the high dielectric signals and their gradual decline with increasing frequency are a result of ionic polarization in the polymer membrane matrix [34].

The electrical conductivity, *σ* (S/cm), is related to the dielectric loss and was further estimated with the relation (3) [35]

(3) $$\sigma =2\pi \;\varepsilon_{0}f\;\varepsilon "$$

where *ε_0_* is the permittivity of the free space and *f* is the applied electric field frequency. 

**Figure 7:**
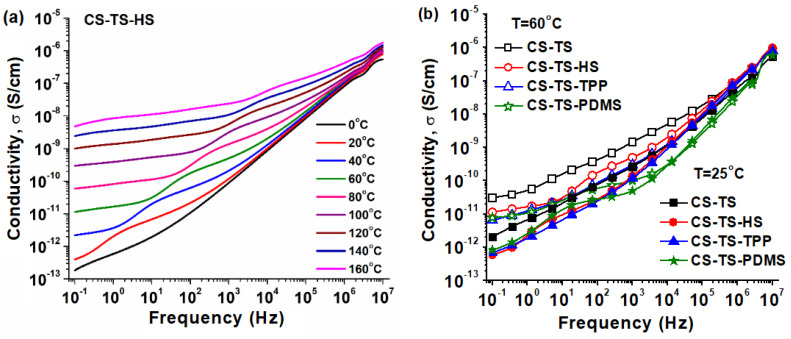
Evolution of the measured conductivity with frequency and temperature for dry membranes: (**a**) CS-TS-HS membrane (**b**) comparison of conductivity behavior of all prepared membranes.

Figure 7 displays the behavior of conductivity with frequency at selected temperatures from 0 to 160 °C for dry membranes. For CS-TS-HS sample (Figure 7a), at low temperature (0 °C), the conductivity exhibits an approximately linear evolution with log frequency and is generally attributed to electronic-type conduction of the bulk membrane [36]. At higher temperatures, deviations from linearity are observed, especially at low frequencies. This region appears in the same frequency range with the linear-type behavior of dielectric constant and dielectric loss. The observed correspondences were previously reported by Pochard et al. [37]. According to literature, the low frequency-independent conductivity plateau could be attributed to the transport of protons through the polymer membrane [36]. Additionally, one may observe an increasing step in *σ*(*f*) spectra that shifts progressively to higher frequencies with temperature. This particular signal called as Maxwell–Wagner–Sillars (MWS) polarization generally appears in heterogeneous systems, at the interface between the components with different dielectric constant (polar sulfate groups within the chitosan matrix) [34,38,39]. As shown in Figure 7a, one may notice that the conductivity values at a frequency of 1 Hz are ranging from 5.9·× 10^−13^ S/cm at *T* = 0 °C to 8.5 ×·10^−9^ S/cm at *T* = 160 °C. The relatively low values correspond to the conductivity of the bulk material and reveal the dielectric-type of the dry CS-TS-HS sample [4]. The *σ*(*f*) spectra of dry CS-TS, CS-TS-TPP, and CS-TS-PDMS systems are presented in Supplementary Materials, Figure S6 and reveal similar dielectric behavior.

The high values of dielectric constant and dielectric loss, especially at low frequencies, suggests the possibility of the use of these materials as PEMs suitable for fuel cell application in a considerable temperature range [36]. Moreover, the frequency evolution of dielectric loss with the slope of −1 could suggest that the segmental dynamics controls the conductivity signal [40]. Since the glass transition of polymer membranes appears above 210 °C, the segmental relaxation should corresponds to the side chain movements from chitosan together with the attached acid groups. In this respect, a comparative evolution of conductivity as function of frequency for all considered dry membranes is displayed in Figure 7b. At lower temperatures (25 °C), the conductivity is mostly electronic, with a small contribution of ionic conductivity localized in the low frequency spectral region (more evident for CS-TS-TTP sample) and generally assigned with proton transfer through different sulfate acid sites. Furthermore, the *σ*(*f*) profiles of cross-linked chitosan membranes furnishes similar behavior probably due to reduced mobility of active sites. The CS-TS-HS, CS-TS-TPP, and CS-TS-PDMS composite membranes presented lower proton conductivity than the CS-TS membrane since the cross-linking process restrict the mobility of polymer segments, hindering the transport of charges [34].

As seen in Figure 7b, at higher temperature (60 °C), the plateau region of proton conductivity is enlarged to higher frequencies, revealing an increased mobility of charge carriers. In this temperature region, the magnitude of σ(f) spectra for CS-TS is still higher than those of cross-linked membranes, due to restrictions imposed by the membrane network. 

The effects of cross-linking with different agents are shown in Table 2, where the proton conductivity values are extracted from *σ*(*f*) spectra of the prepared membranes, choosing the frequency of 1 Hz. 

As seen from Table 2, at 25 °C, σ values of all dry membranes are of the order of 10^−12^ S/cm and are increasing to 10^−11^ S/cm at 60 °C. At 100 °C (see also Figure S7), the values of the conductivity for dry CS-TS-TPP and CS-TS-PDMS are surprisingly similar, revealing that the TPP and PDMS cross-linking agents have comparable effects on the transport of protons through the chitosan membrane. This finding is somewhat surprising since the acid sites of pentasodium tripolyphosphate were expected to enhance the proton conductivity of the membrane. By contrast, the conductivity of CS-TS-HS is at least one order higher than those of CS-TS-TPP and CS-TS-PDMS indicating that the protonation of the amine groups of chitosan by sulfuric acid promotes the protonic conductivity. Thus, among various types of cross-linking agents, the sulfuric acid conducts to superior protonic conductivity at low humidity and high temperatures.

#### 3.8.2. Influence of Water Absorption on the Protonic Conductivity of Membranes

The hydrated membranes were obtained by immersing the samples in distilled water for 1 h at room temperature prior to dielectric spectroscopy measurements. The hydrated CS-TS membrane was not examined, because the water incorporation completely damaged the sample. The dielectric spectra of membranes were collected at different temperatures from 0 to 60 °C, in steps of 5 °C. No reliable spectra were obtained at temperatures higher than 60 °C due to quick evaporation of water.

**Figure 8:**
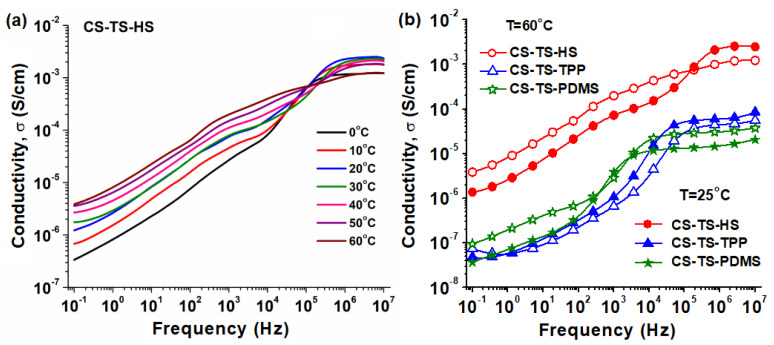
Evolution of conductivity with frequency at different temperatures for hydrated samples: (**a**) CS-TS-HS membrane, (**b**) comparison of conductivity behavior of all cross-linked membranes.

Previous studies have concluded that the hydrophilic sulfonic acid groups from the membranes is primarily responsible for water uptake [2]. As stated above, the water uptake of the studied membranes varies with the cross-linker nature. The resulting conductivity dependences as function of frequency and temperature are shown in Figure 8. *σ*(*f*) spectra similar to that corresponding to hydrated CS-TS-HS membrane were obtained for hydrated CS-TS-TPP and CS-TS-PDMS samples (Figure S8). For CS-TS-HS hydrated membrane (Figure 8a), one may observe dramatically increased *σ*(*f*) magnitudes in the integral frequency range as compared to the dry sample (Figure 7a). Therefore, the membrane saturated with water exhibits conductivity values with about 4 orders of magnitude higher than the corresponding dry membrane, thus suggesting that the conductivity is strongly enhanced by proton migration between polar water molecules. Similar differences between dry and hydrated membranes were previous reported for the standard Nafion 117 [4]. Likewise, the *σ*(*f*) profiles reveal an additional frequency independent conductivity plateau located at high frequencies. 

The comparative *σ*(*f*) spectra of hydrated membranes from Figure 8b and the conductivity values collected in Table 2 reveal that both the low frequency and the high frequency independent frequency plateaus are different in magnitude and their enlargement is depending on membrane composition, water content and temperature. The conductivity of hydrated CS-TS-HS membrane is two orders higher than those of CS-TS-TPP and CS-TS-PDMS because of superior content of sulfuric acid and water uptake capacity (see Section 3.6). Moreover, the conductivities of CS-TS-TPP and CS-TS-PDMS membranes are almost similar, revealing no particular influence of the cross-linking agent. 

According to Figure S9, the conductivity of dry membranes increases linearly with temperature, suggesting that the relaxation dynamics influences the overall conductivity. By contrast, the increase of conductivity with temperature for hydrated samples is noticeably reduced, indicating that the conductivity is primarily highlighted by polar water molecules.

The activation energy for proton transport in chitosan membranes, *E*_σ_, was determined with the Arrhenius-type relation

(4) $$\sigma =\sigma_{0}exp\left(-E_{\sigma }/kT\right)$$

where *σ_0_* is a pre-exponential factor, *k* is the Boltzmann constant, and *T* is the absolute temperature. The activation energy is related to the energy required for proton transport between different polar sites [35].

The activation energy values of the dry membranes are ranging from 53 to 69 kJ/mol. The calculated values are comparable with other systems previously reported [36,41]. Besides, the activation energy for hydrated membranes is much lower than that of dry membranes (ranging from 23 to 31 kJ/mol). According to literature, the values for hydrated membranes suggest that the proton transport occurs primarily via the Grothuss mechanism, i.e., proton migration through hydrogen bond of water molecules by jumping [34,42,43]. 

The measurement of in-plane proton conductivity by four-probes [44,45] method, as described in Section 2.3, was carried out as a confirmation only on the most promising membrane CS-TS-HS that supplied the highest proton conductivity value by dielectric spectroscopy. Such measurement was repeated twice for each temperature in order to have a statistically valid value and hence the result provided is the average between them. Thus, the test through four-probes method (in-plane) at 30 and 60 °C indicated proton conductivity values of 3.0 × 10^−3^ and 6.2 × 10^−3^, respectively.

The results obtained supply a complete overlapping at 30 °C and a good coherence at 60 °C, if compared to the results on the same membrane arising from the dielectric spectroscopy taking into account the substantial differences of the techniques. In Figure S2, the technique used together to the test station and cell for the measurement is described. Such a result confirms the promising capacity of the membrane CS-TS-HS, cross-linked by sulfuric acid.

## 4. Conclusions

Chitosan-sulfated titania composite membranes with appropriate properties for fuel cell applications were produced and the influence of three different cross-linkers—sulfuric acid, pentasodium tripolyphosphate, and polydimethylsiloxane-diglycidyl ether terminated—were studied to obtain their properties. The chemical interaction between chitosan and sulfated titania, as well as the success of the cross-linking reactions, was proved by FTIR structural analysis. The morphological analysis by SEM showed the formation of dense membranes with thicknesses ranging from 31 to 130 μm and uniform dispersion of inorganic filler. The mechanical and thermal measurements indicated that cross-linking processes conducted to tougher materials with thermal stabilities values up to 200 °C. Typical tests usually applied for PEM evaluation, such as water uptake and chemical stability, indicated that the cross-linked membranes developed in the present study can be recommended for fuel cell application. The proton conductivity performances evaluated by dielectric spectroscopy were proven to strongly depend on cross-linker nature and on hydration state of membranes. The most promising membrane was achieved by using sulfuric acid as cross-linker. In addition, according to calculated values of activation energy, the proton transport can occur mainly via the Grothuss mechanism.

## Figures and Tables

**Table 1:** Mechanical properties, water uptake, and stability in oxidative environment and of chitosan-sulfonated titania composite membranes.

Membrane	Mechanical Properties			Water Uptake (%) (24 h)			Weight Loss (%) in Fenton Reagent	
	Tensile Strain (%)	Tensile Stress (MPa)	Young’s Modulus ^2^ (GPa)	25 °C	60 °C	80 °C	1 h	24 h
CS-TS	10.6	24.9	1.54	dissolution			dissolution	
CS-TS-HS	11.8	40.2	1.87	184	172	163	10	15
CS-TS-TPP	- ^1^	15.3	1.46	170	121	135	8	22
CS-TS-PDMS	39.7	50.9	1.01	118	88	92	9	30

^1^ Sample slips in gripping, no break occurred. ^2^ Calculated at 1% strain.

**Table 2:** Values of conductivity at various frequencies for dry and hydrated chitosan-sulfated titania membranes.

Membrane	Conductivity, *σ* (S/cm) at a Frequency of 1 Hz for Dry Membranes			Conductivity, *σ* (S/cm) at Low and High Frequencies for Hydrated Membranes			
	25 °C	60 °C	100 °C	*f* = 1 Hz		*f* = 10^6^ Hz	
				25 °C	60 °C	25 °C	60 °C
CS-TS	6.6 × 10^−12^	5.2 × 10^−11^	3.3 × 10^−9^	-	-	-	-
CS-TS-HS	2.2 × 10^−12^	1.7 × 10^−11^	3.9 × 10^−10^	2.5 × 10^−6^	8.1 × 10^−6^	2.1 × 10^−3^	1.1 × 10^−3^
CS-TS-TPP	1.8 × 10^−12^	1.2 × 10^−11^	7.8 × 10^−11^	5.7 × 10^−8^	5.7 × 10^−8^	5.7 × 10^−5^	4.5 × 10^−5^
CS-TS-PDMS	2.6 × 10^−12^	1.1 × 10^−11^	7.4 × 10^−11^	7.0 × 10^−8^	1.9 × 10^−7^	1.4 × 10^−5^	3.1 × 10^−5^

## Supplementary Materials

The following are available online at https://www.mdpi.com/2073-4360/12/5/1125/s1, Figure S1: 1H NMR spectrum of pristine chitosan (CS); Figure S2: (a) Test station for proton conductivity measurement connected to cell and potentiat-galvanostat; (b) conductivity cell and formula used; Figure S3: Representative surface SEM images of (a) composite chitosan–sulfonated titania membrane (CS-TS) and composite chitosan–sulfonated titania membranes cross-linked with (b) sulfuric acid, (c) pentasodium tripolyphosphate, and (d) polydimethylsiloxane (CS-TS-HS, CS-TS-TPP, and CS-TS-PDMS, respectively); Figure S4: Water uptake kinetics of composite chitosan–sulfonated titania membranes cross-linked with sulfuric acid, pentasodium tripolyphosphate and polydimethylsiloxane (CS-TS-HS, CS-TS-TPP, and CS-TS-PDMS, respectively) at (a) 25 °C, (b) 60 °C and (c) 80 °C; Figure S5: Dielectric constant and dielectric loss evolution with frequency for dry CS-TS, CS-TS-TPP, and CS-TS-PDMS composite membranes; Figure S6 Evolutions of the measured conductivity with frequency for dry (a) CS-TS, (b) CS-TS-TPP, and (c) CS-TS-PDMS composite membranes; Figure S7: The evolution of conductivity with frequency at 100 °C for dry membranes; Figure S8: Evolutions of the measured conductivity with frequency for hydrated CS-TS-TPP and CS-TS-PDMS composite membranes; Figure S9: The evolution of conductivity with temperature at 0.1 Hz for dry and hydrated membranes; and Equation (S1) for Determination of sulfate groups content by back-titration method.
