# Supplementary material for: Chitosan-Sulfated Titania Composite Membranes with Potential Applications in Fuel Cell: Influence of Cross-Linker Nature
Source: Polymers (Basel). 2020 May 14;12(5):1125. doi: 10.3390/polym12051125 (PMC7284654; doi:10.3390/polym12051125)
Supplement: Supplementary file 1 [file polymers-12-01125-s001.pdf]

polymers

Supplementary Materials:

# Chitosan-Sulfated Titania Composite Membranes with Potential Applications in Fuel Cell: Influence of Cross-Linker Nature

**Andra-Cristina Humelnicu [1], Petrisor Samoila [1,*], Mihai Asandulesa [1], Corneliu Cojocaru [1], Adrian Bele [1], Adriana T. Marinoiu [2], Ada Saccà [3] and Valeria Harabagiu [1,*]**

[1] "Petru Poni" Institute of Macromolecular Chemistry, Aleea Grigore Ghica Voda 41A, 700487 Iasi, Romania; humelnicu.andra@icmpp.ro (A.-C.H.); asandulesa.mihai@icmpp.ro (M.A.); cojocaru.corneliu@icmpp.ro (C.C.); bele.adrian@icmpp.ro (A.B.)
[2] National Research and Development Institute for Cryogenics and Isotopic Technologies – ICSI Rm. Valcea, 240050, Romania; adriana.marinoiu@icsi.ro
[3] National Research Council of Italy, Institute for Advanced Energy Technologies "Nicola Giordano" (CNR-ITAE), via S. Lucia sopra Contesse 5, 98126 – Messina, Italy; ada.sacca@itae.cnr.it
* Correspondence: samoila.petrisor@icmpp.ro (P.S.); hvaleria@icmpp.ro (V.H.); Tel.: +40-232-217454

## Determination of molecular weight of chitosan

Molecular weight of the chitosan was determined using an Ubbelohde viscosimeter (Oa capillary, K = 0.005 mm²/s²) at 25 ± 0.05 °C. Chitosan was dissolved in an aqueous solution of 2% acetic acid and 0.2 M sodium acetate (1/1 V/V) to obtain different chitosan concentrations (0.02; 0.04; 0.06; 0.08; 0.1; 0.12 and 0.15 g/dL). To ensure the equilibration of the polymer solution, the solutions were kept for 24 hours at 30 °C. The molecular weight was calculated with Mark-Houwink Equation (S1) by using $k = 13.8 \cdot \times 10^{-5}$ and $a = 0.85$ (*Kasaai, Carbohydrate Polymers 68 (2007) 477–488*).

$$[\eta] = k \cdot M_v^a \quad \text{(S1)}$$

$[\eta] = intrinsic\ viscosity$
$k$ and $a$ = Mark-Houwink parameters
$M_v$ = average viscosity molecular weight

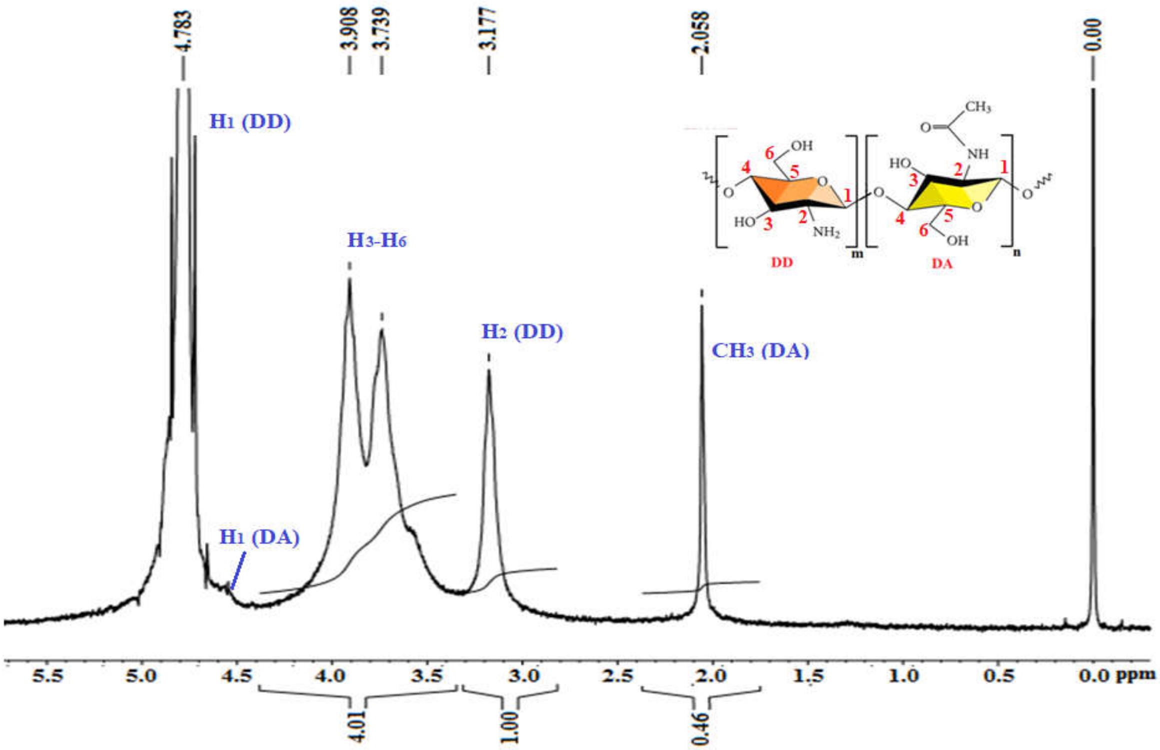


**Figure S1.** $^{1}$H NMR spectrum of pristine chitosan (CS).

The degree of deacetylation (DD) was calculated by using the integral intensity, $I_{CH3}$, of the $CH_3$ residue, and the sum of integral intensities, $I_{H2\text{-}H6}$, of H2, H3, H4, H5 and H6 proton:

$$DD(\%) = (1 - (I_{CH3\text{-}DA}/3)/(I_{H2\text{-}H6}/6))\bullet 100$$

In our particular case, the degree of deacetylation was calculated as follows:

$$DD = (1 - (0.46/3)/(5.01/6)) \times 100 = 81.94\%$$

**Determination of sulfate groups content by back-titration method**

The content of sulfate groups was determined by a common back titration method: Shortly, the sulfated titania particles were suspended in a NaOH solution of known concentration and the suspension was stirred for 2 h for neutralization of sulfate groups and filtrated. The unreacted NaOH was titrated with HCl solution in the presence of phenolphthaleine. The content of sulfate groups/g particles was calculated with Equation S1.

$$Sulfate\ groups\ content\ \left(\frac{mol}{g}\right) = (V_{NaOH} \cdot C_{NaOH} - V_{HCl} \cdot C_{HCl})/m \quad \text{(S2)}$$

where: $V_{NaOH}$ (mL) and $C_{NaOH}$ (Eq/mL) are the volume, respectively the concentration of NaOH solution used to neutralize the sulfate groups; $V_{HCl}$ (mL) and $C_{HCl}$ (Eq/mL) are the volume, respectively the concentration of HCl solution used to titrate the reacted NaOH solution; m (g) is the mass of sulfated titania particles.

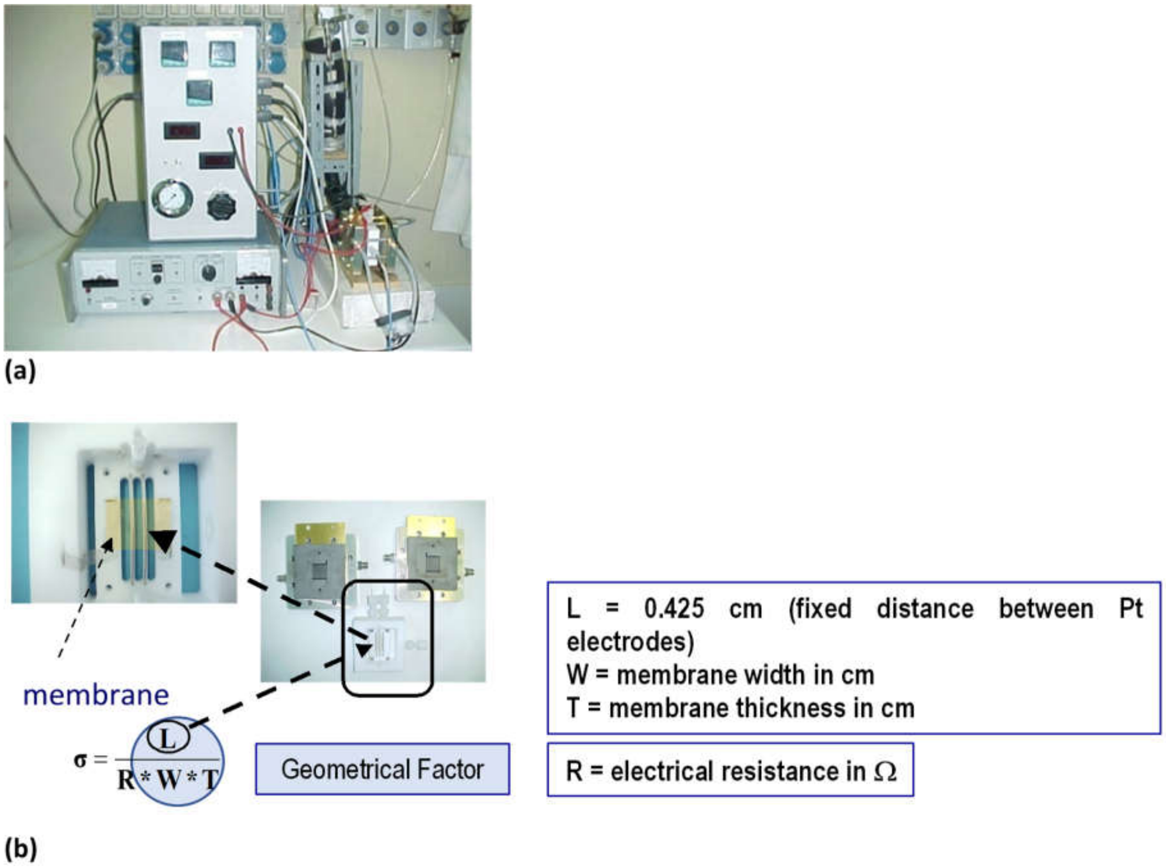


**Figure S2.** (**a**) Test station for proton conductivity measurement connected to cell and potentiat-galvanostat; (**b**) conductivity cell and formula used.

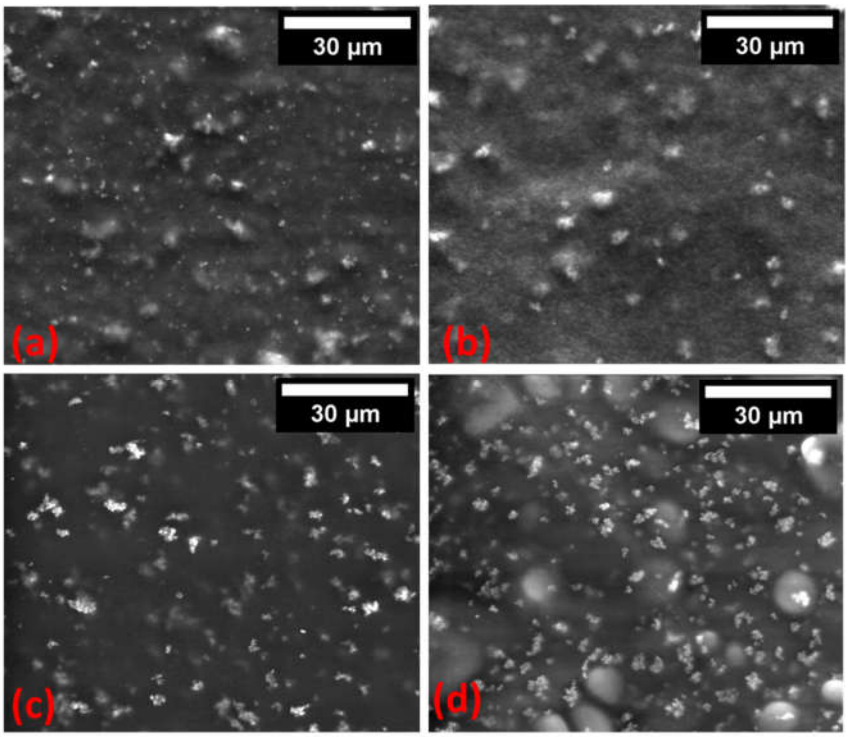

**Figure S3.** Representative surface SEM images of (**a**) composite chitosan – sulfonated titania membrane (CS-TS) and composite chitosan – sulfonated titania membranes cross-linked with (**b**) sulfuric acid, (**c**) pentasodium tripolyphosphate and (**d**) polydimethylsiloxane (CS-TS-HS, CS-TS-TPP and CS-TS-PDMS, respectively).

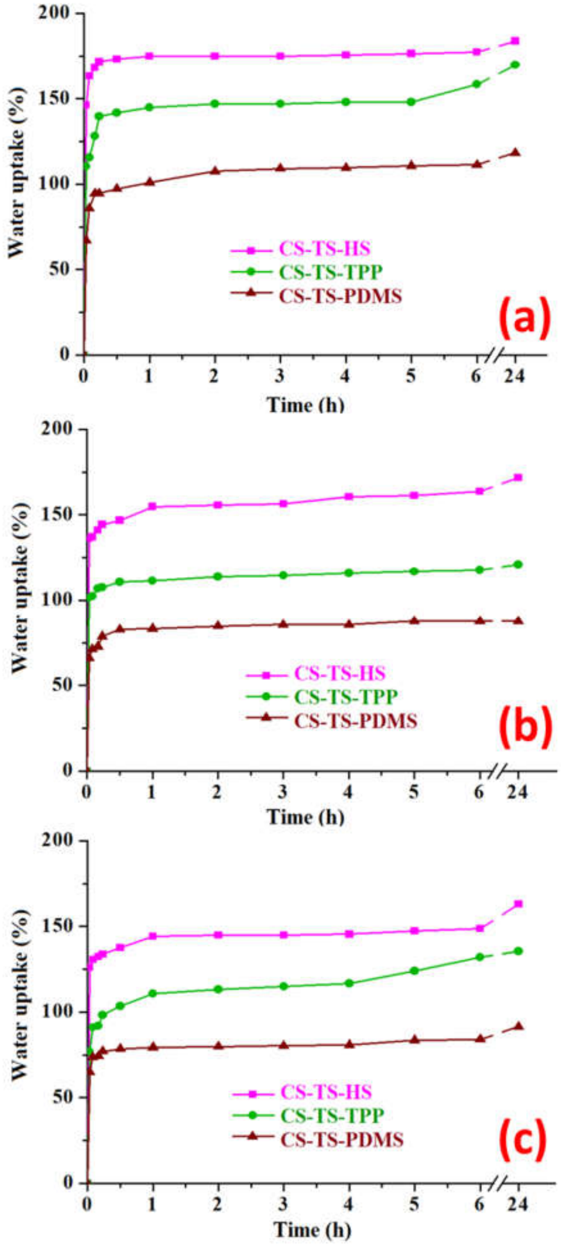


**Figure S4.** Water uptake kinetics of composite chitosan – sulfonated titania membranes cross-linked with sulfuric acid, pentasodium tripolyphosphate and polydimethylsiloxane (CS-TS-HS, CS-TS-TPP and CS-TS-PDMS, respectively) at (**a**) 25°C, (**b**) 60°C and (**c**) 80°C.

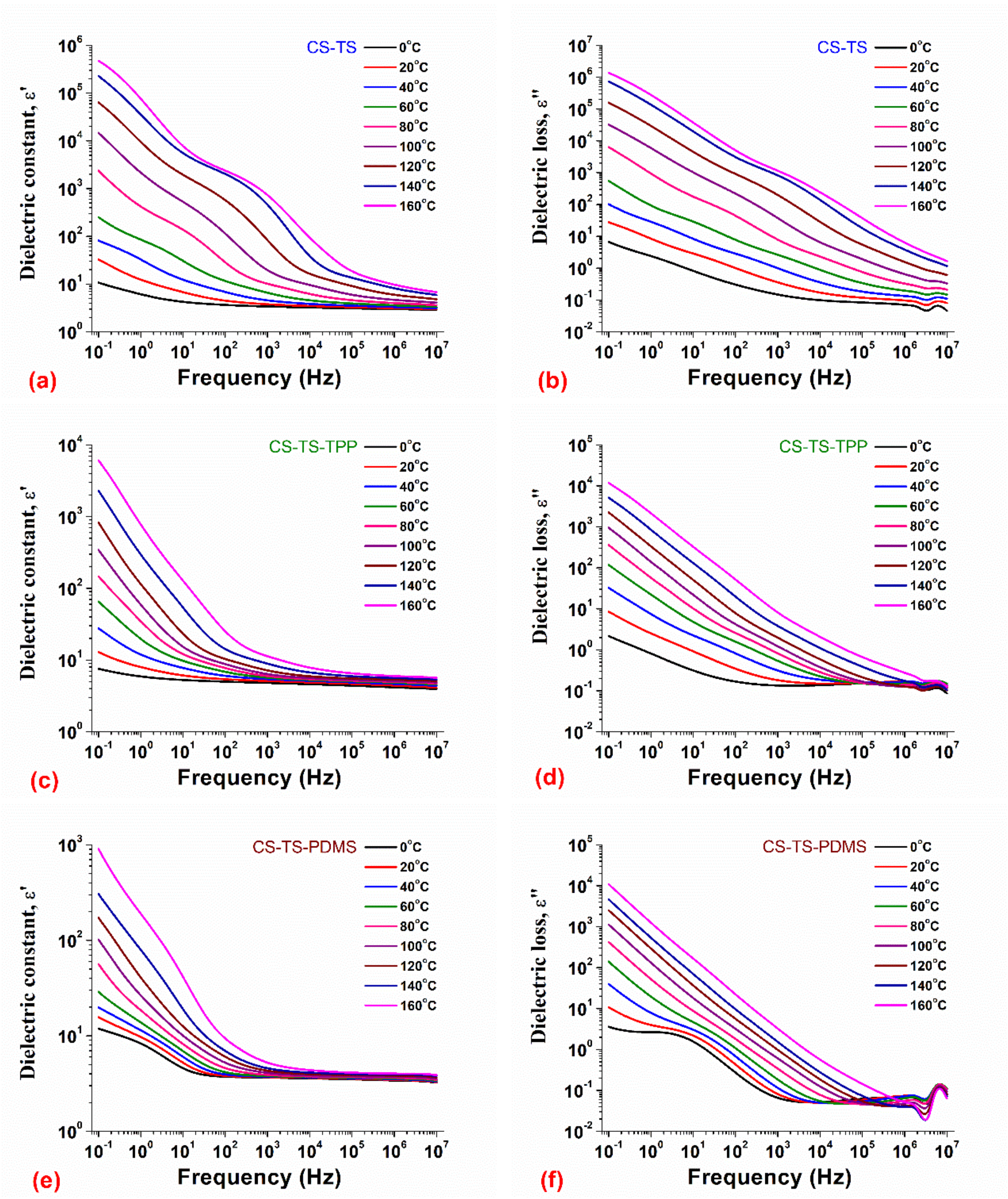


**Figure S5.** Dielectric constant (**a**,**c**,**e**) and dielectric loss (b,d,f) evolution with frequency for dry CS-TS, CS-TS-TPP and CS-TS-PDMS composite membranes.

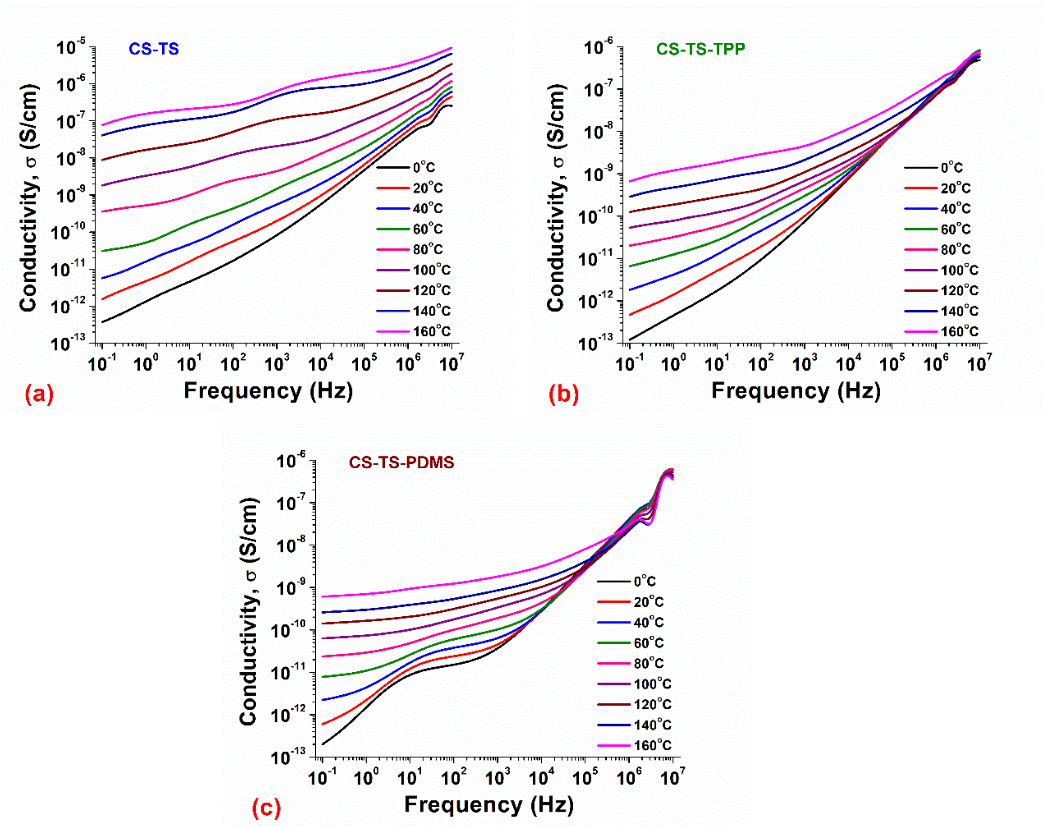


**Figure S6.** Evolutions of the measured conductivity with frequency for dry (**a**) CS-TS, (**b**) CS-TS-TPP and (**c**) CS-TS-PDMS composite membranes.

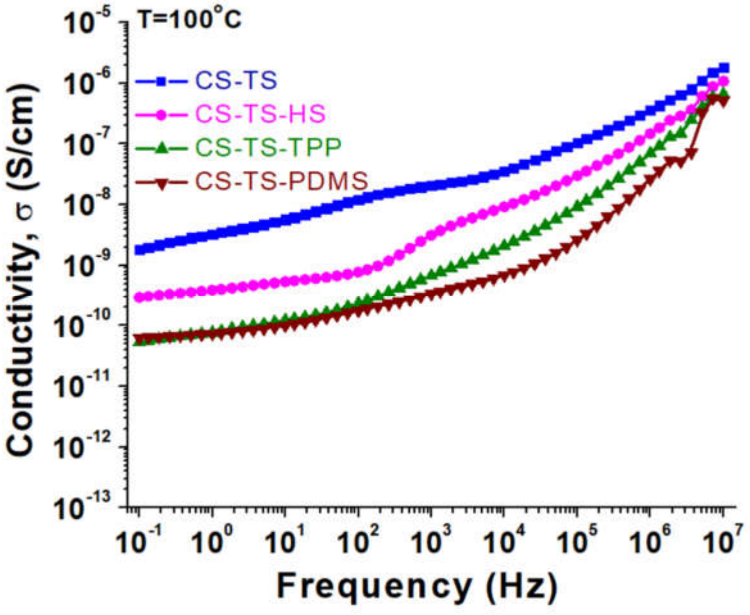


**Figure S7.** The evolution of conductivity with frequency at 100°C for dry membranes.

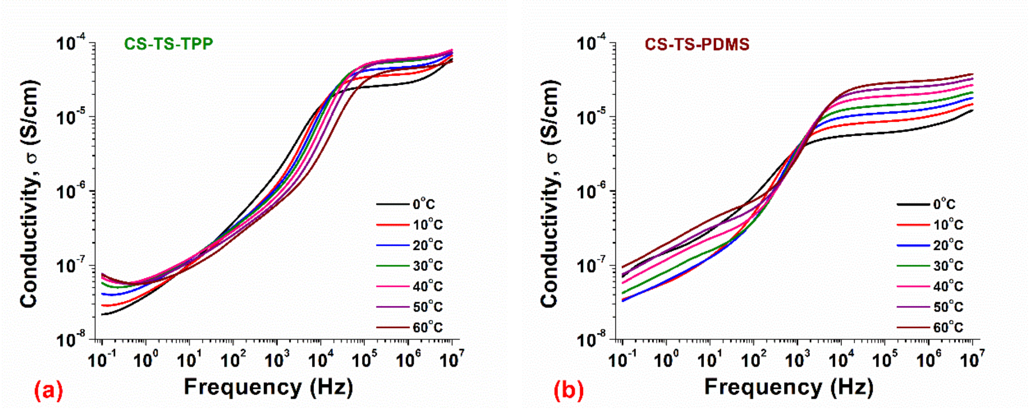


**Figure S8.** Evolutions of the measured conductivity with frequency for hydrated (**a**) CS-TS-TPP and (**b**) CS-TS-PDMS composite membranes.

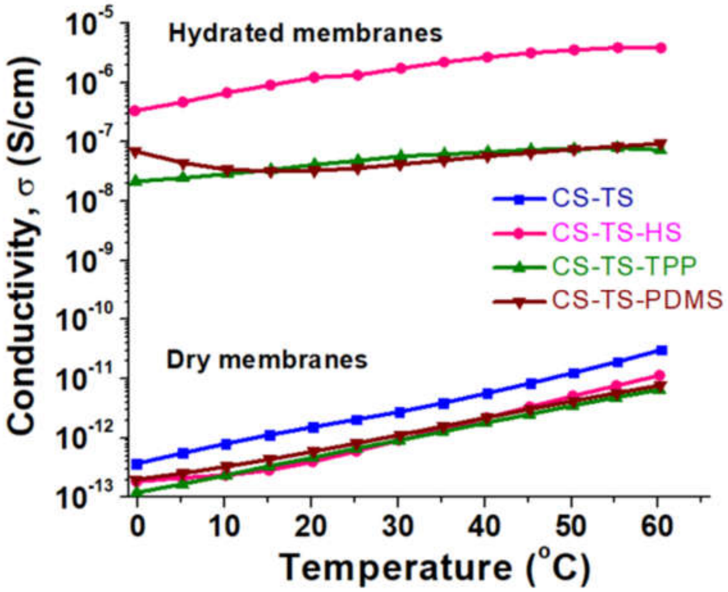


**Figure S9.** The evolution of conductivity with temperature at 0.1 Hz for dry and hydrated membranes.
